# Augmented renal clearance: from pathophysiological mechanisms to clinical implications and research perspectives

**DOI:** 10.1080/0886022X.2026.2696634

**Published:** 2026-07-21

**Authors:** Beiming Xu, Minqiao Chen, Yang Chu

**Affiliations:** Department of Pharmacy, The First Hospital of China Medical University, Shenyang, People’s Republic of China

**Keywords:** Augmented renal clearance, renal diseases, therapeutic drug monitoring, antibiotics dosing, critical illness, clinical implications

## Abstract

**Objectives:**

Augmented renal clearance (ARC) is a critical clinical phenomenon characterized by altered renal excretory function in patients. Although ARC has garnered growing clinical and research attention in recent years, notable gaps remain in its investigation. Inadequate recognition of ARC-related risks may lead to underappreciation of this clinically significant condition in practice. This narrative review synthesizes current evidence on ARC.

**Methods:**

Current evidence on ARC was narratively reviewed, including pathophysiological hypotheses, diagnostic criteria, predictive models, drug-dosing implications and emerging monitoring techniques. Compared with earlier summaries, we place particular emphasis on recent changes in kidney-function assessment for drug dosing, including the distinction between measured glomerular filtration rate (GFR), measured creatinine clearance (CrCl), Cockcroft–Gault estimated CrCl, and contemporary estimated GFR equations.

**Results:**

We discuss the limitations of predictive equations in unstable kidney function, the potential role of repeated short-interval CrCl, bedside hemodynamic assessment, standardized experimental models and multi-omics approaches.

**Conclusions:**

ARC should be regarded as a dynamic pharmacokinetic phenotype requiring timely recognition, drug-specific interpretation, therapeutic drug monitoring and direct or repeated assessment of renal clearance in high-risk patients.

## 1. Introduction

Augmented renal clearance (ARC) is a common clinical phenomenon in the critical care setting, with a reported incidence of 39% to 74% in mixed intensive care unit (ICU) cohorts. The incidence is higher in specific subgroups such as trauma and neurocritical care patients [1]. It is important to note that most critically ill patients with severe trauma or systemic disease are prone to developing renal dysfunction or even acute kidney injury (AKI). However, ARC occurs in a distinct subset of these patients, typically younger individuals with preserved baseline renal function who develop a hyperdynamic circulatory state during ICU admission. In patients with ARC, enhanced renal elimination impairs the clearance of not only endogenous metabolites and electrolytes, but also a broad range of renally excreted medications. This results in abnormally increased drug clearance and subsequent difficulty in achieving therapeutic drug exposure. Consequently, standard dosing regimens often fail to reach pharmacokinetic/pharmacodynamic (PK/PD) targets, complicating clinical management and increasing the risk of subtherapeutic drug concentrations.

Notably, ARC exerts profound clinical consequences. Enhanced renal elimination of medications can lead to subtherapeutic plasma concentrations of renally cleared drugs, which in turn diminishes drug efficacy, contributes to treatment failure, and adversely impacts patient outcomes [2]. Recent studies have documented this effect across multiple drug classes. For antiseizure medications such as levetiracetam, ARC is associated with insufficient serum concentrations despite adherence to standard dosing [3]. For antimicrobial agents including β-lactam antibiotics and vancomycin, ARC is linked to failure to achieve therapeutic exposure and an elevated risk of inadequate infection control [4].

In addition, the underlying pathophysiological mechanisms of ARC remain incompletely elucidated. Potential physiological explanations include increased glomerular filtration driven by elevated renal blood flow, reduced tubular reabsorption, and enhanced tubular secretory activity. All of these may contribute to the accelerated renal elimination of circulating solutes and medications.

Given these challenges, it is necessary to consolidate current knowledge regarding ARC. Although previous reviews have summarized the classical epidemiology, risk factors and antibacterial dosing implications of ARC [5], the present review adds value in three main respects. Firstly, it incorporates recent changes in kidney-function assessment for drug dosing. Secondly, it clarifies that ARC recognition should be viewed as a screening step that prompts closer pharmacokinetic assessment and therapeutic drug monitoring, rather than as a direct indication for uniform dose escalation across all renally cleared drugs. Lastly, it links emerging real-time monitoring technologies, experimental models and omics-based mechanistic studies as translational directions for future ARC research.

## 2. The phenomenon of ARC

### 2.1. Diagnostic criteria and estimation equations

ARC is characterized by abnormally elevated renal function beyond physiological baseline, which results in enhanced elimination of circulating solutes and renally excreted medications. Clinically, ARC is most commonly defined by a measured creatinine clearance (CrCl) > 130 mL/min/1.73 m^2^.

In 1978, Loirat et al. reported that exceptionally high doses of aminoglycosides were required to achieve therapeutic plasma concentrations in burn patients, a finding attributed to markedly increased renal clearance rather than impaired drug efficacy [6]. The clinical importance of ARC was subsequently consolidated by Udy et al. who emphasized its implications for antibacterial dosing in critically ill patients [7].

From the physiological perspective, renal elimination of endogenous solutes and drugs is governed by glomerular filtration, tubular reabsorption, and tubular secretion. Glomerular filtration allows unbound solutes and drugs to pass from the circulation into the Bowman’s capsule. Tubular reabsorption returns certain substances from the tubular lumen back into the systemic circulation, whereas tubular secretion actively transports compounds from peritubular capillaries into the tubular lumen through specific transport systems. For example, aminoglycosides are primarily eliminated through glomerular filtration, while some drugs may be partially reabsorbed along the renal tubules. The increased renal elimination observed in ARC may result from enhanced filtration, altered tubular secretion or reduced tubular reabsorption, which depends on the pharmacokinetic characteristics of the drug involved.

In addition, non-renal factors may also contribute to reduced drug exposure in specific patients. Systemic inflammatory responses or fever can increase metabolic activity and accelerate drug metabolism, while gastrointestinal dysfunction may alter drug exposure through impaired absorption or altered secretion within the gastrointestinal tract. Therefore, when decreased plasma drug concentrations are observed in patients, non-renal mechanisms should also be considered rather than attributing these changes solely to increased renal elimination. This complexity highlights the importance of accurately identifying true ARC in clinical practice.

To date, no single endogenous biomarker can accurately and reliably identify ARC. Timed urinary creatinine clearance (CrCl) and exogenous filtration-marker clearance remain the most robust approaches for ARC assessment. Inulin clearance is the classic reference method for glomerular filtration rate (GFR) measurement, while iohexol and radiolabeled tracer clearance are feasible alternatives in selected settings [8,9]. Newer techniques, particularly transcutaneous GFR monitoring using fluorescent tracer agents, may provide useful tools for dynamic renal function assessment in selected clinical or research settings. For example, relmapirazin has been clinically validated as a fluorescent GFR tracer in comparison with iohexol [10]. In parallel, activatable fluorescent probes have also been explored for evaluating renal biopsy specimens, suggesting that fluorescence-based approaches may have broader applications in renal assessment beyond direct GFR monitoring [11]. However, these methods still require broader validation before routine application in ARC management.

A practical challenge in ARC assessment is the inconsistent use and interpretation of several related but nonequivalent measures of kidney function, including measured GFR, measured CrCl, estimated GFR and estimated CrCl. These measures differ in two key respects: whether renal function is directly measured or estimated using an equation and whether the output reflects GFR or creatinine clearance. This distinction is clinically important because GFR-based and CrCl-based measures are not interchangeable, particularly when renal function assessment is used to guide drug dosing.

Measured GFR reflects the clearance of an exogenous filtration marker and remains the most direct assessment of glomerular filtration. Timed urinary CrCl is more accessible in clinical practice, particularly in catheterized ICU patients, but it may modestly overestimate true GFR because urinary creatinine excretion reflects both glomerular filtration and proximal tubular secretion. Estimated CrCl should also be distinguished from estimated GFR. The Cockcroft–Gault (CG) equation provides an estimate of creatinine clearance rather than GFR and should therefore be interpreted as CG-estimated CrCl. CG-estimated CrCl remains historically important because many drug labels and pharmacokinetic studies were developed on its basis. However, the original equation was derived from a relatively small cohort of adult men using creatinine assays that were not standardized to contemporary reference methods, which limits its accuracy and generalizability in contemporary and heterogeneous patient populations [12]. Therefore, in patients with stable kidney function, contemporary nephrology practice favors validated eGFR equations with body surface area de-indexing for medication-related decisions, while CG-estimated CrCl should be retained primarily when required by drug labels or local dosing protocols.

When kidney function is stable, contemporary nephrology practice increasingly favors validated eGFR equations. The race-free 2021 CKD-EPI creatinine equation, cystatin C equation and combined creatinine–cystatin C equation were developed to improve GFR estimation without race adjustment [13], current guidelines also support the use of cystatin C-based approaches when creatinine is expected to be unreliable [14]. Because CKD-EPI values are indexed to a standard body surface area (BSA) of 1.73 m^2^, this normalization should be removed when patient-specific renal clearance is needed for medication-related decisions. BSA-adjusted eGFR (mL/min) = Indexed eGFR (mL/min/1.73 m^2^) × BSA/1.73. This approach may reduce dosing discordance compared with unadjusted indexed eGFR or CG-estimated CrCl in selected patients [15,16]. The European Kidney Function Consortium (EKFC) equation should also be acknowledged as a contemporary full-age-spectrum approach to GFR estimation [17]. In contrast, the Modification of Diet in Renal Disease (MDRD) equation was developed mainly in patients with reduced kidney function and performs poorly at high GFR ranges. Therefore, it is not well suited for ARC detection.

In patients with unstable kidney function including many ICU patients at risk of ARC, predictive equations are fundamentally limited because they assume a near steady-state relationship between serum creatinine and renal clearance. In this setting, repeated measured CrCl is more informative than a single estimated value. Although 24-h urine collection remains traditional, shorter repeated collections (e.g. 4–8 h) may provide more timely information for recognizing ARC and adjusting drug dosing in catheterized ICU patients. When interpreted together with therapeutic drug monitoring (TDM), these measurements can support real-time assessment of dynamic renal clearance, although collection accuracy and clinical context should still be carefully considered [18,19]. Bedside hemodynamic assessment may provide additional supportive information: point-of-care echocardiography can help identify a hyperdynamic circulatory state, while renal Doppler ultrasound may provide information on renal perfusion and renal vascular resistance [20]. These examinations do not diagnose ARC by themselves, but they may support clinical suspicion and help determine when repeated CrCl measurement and drug-level monitoring are warranted.

The kidney-function assessment methods relevant to ARC identification and drug dosing are summarized in Table 1.

**Table 1.** Kidney-function assessment methods relevant to ARC identification and drug dosing.

Method	Assessment or formula	Relevance to ARC and drug dosing	Limitations and cautions
Measured clearance methods: exogenous-marker mGFR and timed urinary CrCl	Exogenous-marker clearance uses inulin, iohexol, or radiolabeled tracers. For timed urinary CrCl: $\text{CrCl}=\frac{U_{\text{Cr}}\times V}{S_{\text{Cr}}}$	Exogenous-marker mGFR is a reference approach when high precision is required. Timed urinary CrCl is the most practical clinical method for confirming ARC; shorter repeated collections may be useful in catheterized ICU patients when rapid reassessment is needed.	Exogenous-marker methods are rarely available for urgent ICU dosing. CrCl may modestly overestimate true GFR because creatinine is also secreted by renal tubules, and urine collection error may affect accuracy.
Cockcroft–Gault (CG) equation	$\text{CrCl}=\frac{(140-\text{Age})\times \text{Wt}\times K}{72\times S_{\text{Cr}}}$ where *K* = 1.0 for males and 0.85 for females when SCr is in mg/dL.	Provides CG-estimated CrCl in mL/min and remains the historical basis for many drug labels and pharmacokinetic studies.	Estimates CrCl, not GFR; derived from older data using non-standardized creatinine; may be unreliable at high clearance values.
Contemporary eGFR equations: CKD-EPI 2021 and EKFC	CKD-EPI 2021 includes creatinine-based, cystatin C-based, and combined creatinine-cystatin C equations. EKFC is a full-age-spectrum eGFR approach. For dosing: BSA-adjusted eGFR = indexed eGFR×BSA/1.73	Useful in stable kidney function. Cystatin C or combined creatinine-cystatin C equations may improve estimation when creatinine is unreliable. BSA de-indexing converts indexed eGFR to an individual value in mL/min for medication-related decisions.	Not a substitute for measured CrCl in unstable ICU patients. ARC-specific validation remains limited, especially for very high clearance states.
Modification of Diet in Renal Disease (MDRD) equation	Historical creatinine-based eGFR equation developed mainly in patients with reduced kidney function.	Previously used for CKD staging.	Poor performance at high GFR ranges; unsuitable for ARC detection.

**Abbreviations:** ARC: augmented renal clearance; BSA: body surface area; CG: Cockcroft–Gault; CKD-EPI: Chronic Kidney Disease Epidemiology Collaboration; CrCl: creatinine clearance; eGFR: estimated glomerular filtration rate; EKFC: European Kidney Function Consortium; GFR: glomerular filtration rate; ICU: intensive care unit; MDRD: modification of diet in renal disease; mGFR: measured glomerular filtration rate; SCr: serum creatinine; UCr: urinary creatinine concentration; V: urine flow rate; Wt: body weight.

### 2.2. Biomarkers related to ARC

Beyond creatinine, several alternative biomarkers for estimating GFR have been investigated. Cystatin C is a low-molecular-weight cysteine protease inhibitor produced at a relatively constant rate by nucleated cells. Compared with creatinine, it is less dependent on muscle mass and is not actively secreted by renal tubules [21]. In the context of ARC, cystatin C should be interpreted in two complementary ways: as an independent filtration marker and as an input for cystatin C-based or combined creatinine–cystatin C equations.

The 2021 CKD-EPI cystatin C and creatinine–cystatin C equations may improve GFR estimation when creatinine is unreliable [13], and current guideline recommendations also support the use of cystatin C-based approaches in selected clinical scenarios [14]. However, cystatin C is not ARC-specific and may be influenced by thyroid dysfunction, systemic inflammation and corticosteroid exposure. Thus, cystatin C can refine renal function estimation in selected patients, but it cannot replace measured clearance in unstable ICU patients.

Proenkephalin (PENK) has also been proposed as a novel biomarker for renal function assessment [22]. PENK is stably expressed in the circulation, does not bind to plasma proteins, and is freely filtered by the glomerulus without tubular secretion or reabsorption, rendering its plasma concentration closely reflective of true GFR. Experimental and clinical studies have demonstrated that circulating PENK levels correlate strongly with measured GFR and are independently associated with the occurrence, severity, and prognosis of AKI across diverse patient populations. Nevertheless, despite its promising physiological characteristics, the clinical implementation of PENK remains constrained by the limited size of validation cohorts, lack of large-scale prospective studies, and absence of standardized assay methodologies, which collectively hinder its widespread adoption.

Despite these advances, serum creatinine remains the predominant biomarker for estimating GFR in clinical practice because of its widespread availability, low cost, and convenience. Although the limitations of creatinine are well recognized (e.g. the dependence on muscle mass, dietary intake, and tubular secretion), no alternative biomarker has yet demonstrated sufficient accuracy, feasibility and cost-effectiveness to justify replacing creatinine in routine ARC screening. Therefore, future biomarker development should prioritize not only a strong correlation with renal function but also clinical practicality, including noninvasive measurement, standardization and economic feasibility.

## 3. Mechanisms associated with ARC

The precise mechanisms of ARC remain incompletely elucidated, though several hypotheses have been proposed to explain its pathophysiology, including the systemic inflammatory response syndrome (SIRS), renal functional reserve (RFR) theory, and brain-kidney crosstalk theory.

### 3.1. Systemic inflammatory response syndrome

ARC has been frequently observed in critically ill patients with systemic inflammatory states such as sepsis associated with SIRS. Reports have documented that a subset of human septic patients exhibit increased renal blood flow [23], and experimental animal studies have shown that renal blood flow can be preserved or even increased under hyperdynamic conditions [24]. However, ARC cannot be simply attributed to SIRS itself. Rather, current evidence suggests that ARC is more likely to occur in a subset of critically ill patients with preserved baseline renal function who develop a hyperdynamic circulatory state, particularly in the context of sepsis, trauma, or major burns. In these patients, systemic inflammation may be accompanied by increased cardiac output and, in some cases, by elevated renal blood flow and glomerular filtration, thereby promoting enhanced clearance of solutes and drugs. Nevertheless, this response is not universal, and sepsis-associated renal hemodynamics are heterogeneous across patients.

SIRS is closely linked to sepsis. Under traditional definitions, sepsis was defined as SIRS in the presence of suspected infection, severe sepsis as sepsis with organ dysfunction and septic shock as severe sepsis with persistent hypotension despite adequate fluid resuscitation. Current guidelines [25] recommend initial fluid resuscitation with 30 mL/kg isotonic crystalloids for patients with severe sepsis or septic shock, followed by vasopressors and inotropes when fluid responsiveness is inadequate. These hemodynamic interventions may increase cardiac output and renal blood flow in certain patients, potentially contributing to supraphysiologic glomerular filtration and predisposing susceptible individuals to ARC.

Therapeutic interventions may further modulate this process, with highly variable effects. Fluid resuscitation and restoration of effective circulatory volume may support renal perfusion in some patients. However, vasoactive agents cannot be assumed to uniformly increase renal blood flow or glomerular filtration. Experimental and clinical data indicate that the renal effects of vasoactive agents depend on the underlying hemodynamic state. For example, epinephrine has been shown to reduce renal blood flow in experimental hyperdynamic sepsis, whereas norepinephrine may improve renal perfusion in some patients by restoring perfusion pressure, but not by directly increasing renal blood flow in all cases [26].

Collectively, the most plausible interpretation is that systemic inflammation contributes to ARC indirectly. The development of ARC depends on the combined effects of the inflammatory response, hyperdynamic circulatory state, and clinical resuscitation interventions.

### 3.2. Renal functional reserve theory

RFR [27] is defined as the difference between baseline GFR and stimulated GFR measured after a physiological challenge, classically a protein load. RFR reflects the kidney’s latent capacity to increase glomerular filtration in response to metabolic or hemodynamic stress and is considered a marker of renal adaptability.

The concept of RFR was first systematically described by Bosch et al. who demonstrated that protein loading significantly increased GFR in healthy individuals, revealing a previously unutilized filtration capacity [28]. Under normal physiological conditions, the kidneys operate below their maximal filtration capacity. However, RFR can be activated during specific physiological states (e.g. high-protein intake, pregnancy, solitary kidney) or pathological conditions (e.g. diabetes, hypertension, AKI), leading to renal hyperfiltration.

Multiple factors modulate RFR, including age, baseline renal function and comorbidities [29]. Younger individuals generally exhibit a greater RFR [30], which is consistent with epidemiological observations identifying young age as a major risk factor for ARC. Conversely, conditions such as pregnancy, cardiorenal syndrome, kidney donation, and chronic hyperfiltration states (e.g. diabetes or hypertension) are associated with reduced RFR [31]. Musso et al. reported that young patients with HIV infection exhibited significantly reduced renal functional reserve compared with seronegative healthy controls [32].

Importantly, increased renal blood flow or GFR does not necessarily translate into enhanced clearance of drugs or other solutes. ARC represents a clinical condition characterized by enhanced renal elimination of circulating solutes and renally cleared drugs. This process is influenced not only by glomerular filtration but also by additional determinants such as renal tubular secretion, drug transporters, systemic hemodynamics, and pathophysiological changes associated with critical illness. Therefore, the presence of increased filtration capacity alone does not inevitably result in augmented drug clearance.

Nevertheless, preserved or enhanced RFR may provide a physiological substrate that allows the kidney to increase filtration capacity when exposed to systemic hyperdynamic circulation or inflammatory stress. In critically ill patients, particularly those with systemic inflammatory responses, the interaction between preexisting RFR and disease-related hemodynamic alterations may facilitate the development of ARC [33]. However, direct evidence linking RFR activation to ARC remains limited, further studies are required to clarify this relationship.

### 3.3. Brain–kidney crosstalk theory

The brain–kidney crosstalk theory has been proposed to explain bidirectional interactions between the central nervous system (CNS) and renal function, particularly in the context of AKI [34]. AKI may adversely affect the brain through systemic inflammation, increased blood–brain barrier permeability, metabolic acidosis and osmotic disturbances, which can lead to cerebral edema and neurological dysfunction. Conversely, CNS injury can disrupt renal homeostasis through neurohumoral and autonomic pathways.

Davenport et al. proposed that bidirectional communication between the brain and kidneys occurs *via* neural, humoral and immune mechanisms to maintain fluid and electrolyte balance [35]. This communication becomes dysregulated in pathological states such as AKI and CKD, resulting in clinically significant disturbances.

ARC is frequently observed in patients with traumatic brain injury (TBI), which highlights the potential role of brain-kidney crosstalk in the pathophysiology of ARC. TBI management typically involves hypertonic saline administration and norepinephrine infusion to optimize cerebral perfusion pressure [36]. Udy et al. reported a high incidence of ARC during cerebral perfusion pressure management in TBI patients [37]. Dias et al. further explored this interaction by demonstrating that preserved autoregulation of cerebral and renal blood flow maintains stable perfusion despite variations in arterial blood pressure and intracranial pressure. Using the pressure reactivity index derived from intracranial pressure and arterial blood pressure, they found a significant association between preserved cerebrovascular reactivity and elevated CrCl, suggesting a link between intact cerebral autoregulation and ARC occurrence [38].

Recent pharmacokinetic studies have demonstrated that ARC in TBI patients can substantially increase the renal elimination of several renally cleared medications. For example, levetiracetam has been shown to exhibit significantly reduced plasma concentrations in TBI patients with enhanced renal clearance [3]. In addition, clinical studies in brain-injured patients with ventilator-associated pneumonia have reported that ARC is associated with increased clearance of β-lactam antibiotics and a higher risk of antibiotic treatment failure due to subtherapeutic drug exposure [39].

Udy et al. also proposed that ARC in TBI patients may be associated with elevated atrial natriuretic peptide levels and a hyperdynamic cardiovascular state [40]. Khalid et al. suggested that TBI-associated autonomic dysfunction may disrupt brain–kidney signaling and renal perfusion regulation, thereby promoting enhanced renal clearance [41]. Younger age remains the only independent predictor of ARC in TBI patients [42], further supporting the interaction between physiological reserve and ARC development. Overall, the mechanistic basis of brain–kidney crosstalk in ARC warrants further investigation.

**Figure 1.**
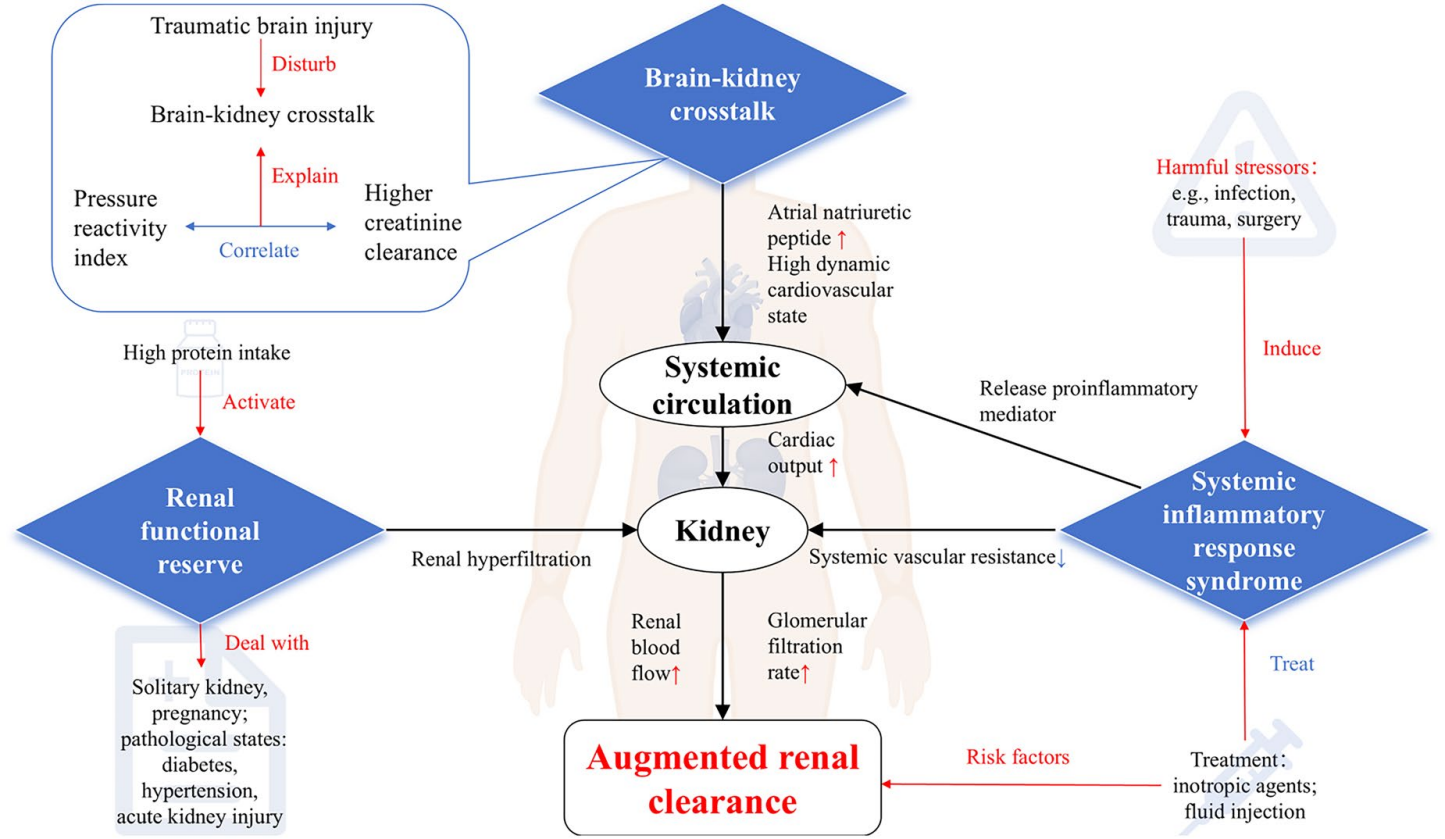
Mechanisms underlying ARC. The schematic diagram illustrates three proposed mechanisms contributing to the development of ARC.

Figure 1 summarizes the three proposed mechanisms underlying ARC.

## 4. Relationship between ARC and clinical practice

### 4.1. ARC predictive scoring models

Several predictive scoring models have been developed to improve the clinical identification of ARC. Barletta et al. established the Augmented Renal Clearance in Trauma Intensive Care (ARCTIC) model for trauma patients. A score of more than 6 in this model indicates an 82% probability of ARC in this population [43]. The model was initially validated in trauma patients and was later shown to be applicable in mixed medical-surgical ICU cohorts [44].

Gijsen et al. proposed an ARC predictor that includes six clinical variables. These variables are ICU stay duration, age, sex, serum creatinine concentration, trauma history and cardiac surgery history. The model is designed to facilitate the early identification of patients at high risk of ARC [45]. Julie et al. compared the ARCTIC model with the ARC predictor in clinical practice. They reported that the ARC predictor had slightly better predictive performance and both tools were considered clinically reliable for ARC screening [46].

Udy et al. also developed an alternative ARC scoring system based on readily available clinical variables to estimate the risk of ARC in critically ill patients [47]. Subsequently, Kanna et al. demonstrated that lowering the cutoff threshold of this scoring system to 5 improved its sensitivity for ARC detection [48]. In addition, Ryusei Mikami et al. developed the Japanese augmented renal clearance (JPNARC) score, which incorporates age, sex, serum creatinine, and ICU admission diagnosis, including trauma and central nervous system disorders [49]. This region-specific model highlights the potential value of developing ARC prediction tools tailored to specific populations, such as critically ill patients in China.

Importantly, the primary clinical value of these predictive scoring models lies in early identification and risk stratification of patients who may develop ARC, rather than serving as a direct guide for automatic drug dose escalation. Moreover, the clinical implications of ARC vary depending on the pharmacokinetic properties of individual medications. Drugs that are predominantly eliminated through renal filtration are more likely to exhibit reduced systemic exposure in patients with ARC. Therefore, predictive scoring systems should be regarded as screening tools that help identify patients who may require closer pharmacokinetic monitoring and individualized dosing, rather than as standalone determinants for empiric dose escalation.

### 4.2. Risk factors associated with ARC

The variables included in ARC predictive models largely reflect recognized clinical risk factors. These determinants can be broadly grouped into patient-related factors, disease-related conditions, and hemodynamic or treatment-associated influences [50]. Younger age has consistently emerged as an independent predictor of ARC across diverse ICU settings. Both univariate and multivariate analyses have demonstrated that trauma is strongly associated with ARC [51]. Male sex and lower illness severity scores have also been reported as contributing factors.

Conditions associated with systemic inflammation and hyperdynamic circulation, including febrile neutropenia [52], sepsis [53] and intracerebral hemorrhage, are frequently observed in patients with ARC. In these contexts, enhanced cardiac output and renal blood flow are thought to contribute to elevated glomerular filtration. Moreover, therapeutic interventions including aggressive fluid resuscitation and vasopressor administration may further influence renal perfusion and drug elimination. Overall, ARC appears most frequently in patients with hyperdynamic physiology superimposed on specific host characteristics, underscoring the importance of careful monitoring in high-risk populations.

From a practical standpoint, these risk factors should prompt not only laboratory screening but also hemodynamic assessment. Point-of-care echocardiography and renal Doppler ultrasound are increasingly available in ICUs and can help characterize high-output circulation, fluid responsiveness, renal perfusion, and renal vascular resistance [20]. Their role is supportive rather than diagnostic: a hyperdynamic ultrasound profile should increase suspicion for ARC and lower the threshold for repeated measured CrCl and therapeutic drug monitoring.

### 4.3. Special populations at high risk of ARC

Special populations closely associated with ARC require particular attention from healthcare professionals. ICU patients are often exposed to interventions that may induce ARC, including prolonged fluid resuscitation and vasopressor administration. ARC research in ICU patients is relatively complex. The main reasons are the severity of critical illness, hemodynamic instability and high incidence of postoperative or nosocomial infections [54].

Dhont et al. emphasized the need for increased awareness of ARC in critically ill pediatric patients [55]. Unlike adults, pediatric patients undergo rapid growth and organ maturation, which complicates the assessment of renal function and ARC identification. Heggen et al. found that male sex and mechanical ventilation were significantly associated with ARC in pediatric populations [56]. Although the modified Schwartz formula is commonly used to estimate renal function in children [57], Heggen et al. cautioned against overreliance on its accuracy for detecting ARC.

The relationship between ARC and obstetric patients also warrants consideration. Pregnancy represents a physiological state characterized by progressive increases in baseline GFR due to renal hyperfiltration. Although pregnancy itself is not an independent risk factor for ARC, studies suggest that ARC is prevalent among critically ill obstetric patients admitted to the ICU [58,59].

### 4.4. The impact of ARC on drug therapy

The impact of ARC on pharmacotherapy should be interpreted in a drug-specific manner. ARC does not automatically mandate indiscriminate dose escalation. Its clinical relevance depends on the extent of renal elimination, protein binding, tubular secretion or reabsorption, tissue penetration, therapeutic window and the pharmacodynamic target of each drug. Therefore, recognition of ARC should trigger closer assessment of renal clearance and drug exposure rather than a fixed dosing rule.

For patients with stable kidney function, renal dosing decisions should increasingly consider patient-specific, non-indexed GFR when this is compatible with drug-specific dosing guidance. This means converting an indexed CKD-EPI value to mL/min by adjusting for body surface area and considering cystatin C or combined creatinine-cystatin C estimates when creatinine is likely to be misleading [13–16]. CG-estimated CrCl may still be used when a drug label or local protocol explicitly relies on it, but clinicians should recognize that it estimates CrCl rather than GFR and may be unreliable at the high clearance values typical of ARC [12]. In high-clearance ARC states, CG-estimated CrCl may misclassify the actual magnitude of renal clearance and should therefore be interpreted cautiously.

For critically ill patients with unstable kidney function, estimated equations should not be used as the sole basis for dose adjustment. In catheterized ICU patients, performing repeated timed urinary CrCl measurements over 4–8 h, combined with therapeutic drug monitoring, represents a practical strategy to recognize ARC and adapt drug dosing in real time. This approach allows clinicians to capture rapid fluctuations in renal clearance that may be missed with longer collection intervals [18,19]. Bayesian dose optimization, when available, may further improve the link between measured renal clearance, observed drug concentrations, and target exposure.

Antibiotics remain the best-studied drug class in ARC, and surveys suggest that awareness of ARC-related antimicrobial dose adjustment remains insufficient in clinical practice [60]. Subtherapeutic exposure to β-lactam antibiotics and vancomycin is common during early critical illness, and ARC is a major contributor to failure to reach PK/PD targets [61–63]. Standard dosing regimens may, therefore, be inadequate for selected renally eliminated antibiotics, particularly when ARC is severe or persistent. However, dose optimization should be guided by PK/PD principles and TDM whenever feasible, rather than by empirical dose escalation alone.

Gatti et al. showed that rapid attainment of appropriate PK/PD targets improves clinical efficacy and may reduce β-lactam resistance [64]. In this context, prolonged or continuous infusion, loading doses, TDM-guided maintenance dosing and dose maximization within accepted safety ranges are more rational than simply increasing the total daily dose. For meropenem, prolonged or continuous infusion can increase the probability of target attainment and may be preferable to dose escalation alone [65,66]. Combination therapy, such as aminoglycoside plus β-lactam regimens, may be considered in selected critically ill patients when microbiological risk is high, but this strategy requires careful toxicity monitoring [67].

The clinical consequences of ARC are not limited to antibiotics. Levetiracetam and linezolid illustrate that ARC or ARC-like hyperclearance can affect neurocritical care and anti-infective therapy even when non-renal factors also contribute to exposure variability [68]. Future studies should therefore expand beyond antibacterial agents and evaluate sedatives, antiseizure drugs, anticoagulants and cardiovascular medications for which underexposure may have immediate clinical consequences. In practice, severe or persistent ARC should prompt early TDM whenever an assay is available, especially for drugs with narrow therapeutic windows or for drugs in which failure to attain target exposure may have serious clinical consequences. Well-designed prospective cohorts are still needed to define drug-specific thresholds and dosing algorithms.

## 5. Future research directions concerning ARC

The clinical impacts of ARC have attracted increasing attention in critical care practice, as the condition is closely associated with subtherapeutic drug exposure and poor clinical outcomes in affected patients. However, the underlying pathophysiological mechanisms of ARC have not yet been fully clarified in current research. In fact, the exploration of ARC mechanisms is faced with multiple practical challenges in both clinical and basic research. ARC presents as a rapidly changing clinical state in patients, which makes it easy to miss the optimal time for detection and sample collection in clinical practice. Relevant clinical samples from ARC patients are also difficult to obtain for research due to the above characteristics. In addition, there is a severe lack of standardized and clinically relevant experimental animal models for ARC research at present. These limitations have slowed mechanistic research and the development of targeted interventions for ARC. Optimizing and establishing standardized ARC animal models and conducting mechanistic research based on omics technologies are two possible research directions to further elucidate the pathogenesis of ARC.

### 5.1. Animal models: challenges in establishing standardized ARC models

Dhondt et al. explored the feasibility of establishing an ARC piglet model induced by fluid therapy [69]. The renal anatomy and physiology of piglets are highly similar to those of humans. This characteristic makes the model suitable for simulating ARC in pediatric patients. The study confirmed that continuous fluid infusion can induce ARC in piglets. However, this model has several limitations in clinical applicability. It cannot fully reproduce the complex pathological conditions of critically ill patients, such as severe inflammation and multiple organ dysfunction.

Small animal models have obvious advantages over large animal models such as piglets. They have lower research costs and higher experimental flexibility, which are conducive to large-scale experimental studies. ARC is closely associated with sepsis, TBI and inflammatory responses as mentioned earlier. Yang et al. explored the potential of constructing small animal models related to ARC from multiple aspects [70]. The models studied include sepsis models, burn models, subarachnoid hemorrhage models and TBI models.

High-protein diets or other interventions can be used to enhance renal functional reserve in animals. This is another potential method to induce ARC in animal models. Trauma has been confirmed to be a clinical condition associated with ARC and renal hyperfiltration in both observational and experimental studies. The role of trauma in the establishment of ARC animal models needs to be further explored. The current small-animal models of ARC are not yet fully characterized. More systematic and in-depth research is needed to improve these models.

### 5.2. Omics technologies: multi-omics approaches to elucidate ARC mechanisms

Omics technologies refer to systematic approaches for investigating specific molecular families within biological systems. Owing to their high analytical sensitivity and throughput, omics technologies have been widely applied in biomedical research. Currently, four major omics disciplines dominate the field: genomics, transcriptomics, proteomics, and metabolomics.

The high sensitivity of omics technologies helps to identify and validate disease-related biomarkers. Omics approaches have been used in cancer research to identify and evaluate biomarkers for prostate cancer [71]. Complex omics datasets can be combined with machine learning and pathway analysis. This integration can significantly improve the efficiency of data interpretation. Some research methods such as random forest analysis have been used to construct high-accuracy prediction models [72].

The flow of biological information generally follows the order of DNA to RNA to proteins and finally to metabolites. This flow corresponds to the four major omics research layers. Proteomics and metabolomics are more dynamic and tissue-specific than genomics and transcriptomics. They can thus reflect disease phenotypes more accurately. A comprehensive understanding of disease mechanisms usually requires multi-omics integration. This integration needs to be based on genomic data as the core.

Omics technologies have shown unique application value in nephrology research. The applications include mechanistic studies of membranous nephropathy, prediction of CKD incidence and research on AKI [73]. The application of omics approaches to the study of ARC pathophysiology is a promising research direction. Previous studies have combined metabolomics and transcriptomics to explore ARC mechanisms. These studies suggest that ARC is associated with inflammation-related gene regulation and changes in genes related to glomerular capillary wall permeability [74]. These findings supplement the existing mechanistic theories of ARC. Further clinical and experimental validation is still needed to confirm these results.

## 6. Summary and perspectives

Augmented renal clearance represents a complex and dynamic pharmacokinetic phenotype rather than a simple increase in a serum creatinine-based estimate of kidney function. Although ARC is traditionally defined by elevated measured CrCl, its clinical meaning extends beyond a single clearance threshold and reflects the combined influence of systemic hemodynamics, renal functional reserve, tubular transport processes and drug-specific pharmacokinetic properties. From a drug-dosing perspective, it is particularly important to distinguish measured GFR, measured CrCl, estimated CrCl and indexed or non-indexed eGFR. In this context, the patient’s individual renal clearance expressed in mL/min is often more relevant than a body surface area-normalized value expressed in mL/min/1.73 m^2^.

From a mechanistic perspective, existing hypotheses (e.g. systemic inflammatory response, renal functional reserve and brain–kidney crosstalk) should not be viewed as mutually exclusive. Instead, they represent complementary components of an integrated physiological response to critical illness. The interaction between hyperdynamic circulation and preserved renal adaptive capacity appears to be central to ARC development, while emerging evidence suggests that tubular transport mechanisms and drug-specific properties may further modulate drug clearance. Future studies should therefore aim to move beyond isolated mechanistic models toward a systems-level understanding of ARC.

Clinically, ARC poses a significant challenge for pharmacotherapy, particularly for drugs with narrow therapeutic windows or predominant renal elimination. One important change in perspective since earlier reviews is the move away from uncritical reliance on Cockcroft–Gault estimated CrCl and MDRD toward contemporary CKD-EPI 2021 and EKFC equations in stable patients, with body surface area de-indexing for medication-related decisions and greater use of cystatin C when creatinine is unreliable. In unstable ICU patients, however, no estimating equation is a substitute for repeated measured CrCl, therapeutic drug monitoring and clinical pharmacokinetic judgment. The discrepancy between estimated renal function and actual drug clearance further underscores the need for more reliable and dynamic monitoring strategies. Although TDM remains central to individualized treatment, its implementation may be limited by assay availability and delayed feedback. The integration of real-time GFR monitoring technologies with pharmacokinetic modeling may therefore represent a promising direction for optimizing drug dosing in patients with ARC.

Importantly, current research has predominantly focused on antimicrobial agents, whereas the impact of ARC on non-antibiotic drugs remains underexplored. This gap may have important clinical implications, particularly in neurocritical care and cardiovascular pharmacotherapy, where precise drug exposure is essential. Expanding research beyond antibiotics will be crucial for a more comprehensive understanding of ARC-related pharmacokinetic alterations.

From a translational research perspective, the lack of standardized and reproducible experimental models remains a major barrier to mechanistic investigation. Although existing animal models partially replicate ARC-associated conditions, they fail to capture the dynamic and multifactorial nature of the syndrome. The integration of multi-omics approaches with well-characterized experimental models may provide new insights into the molecular basis of ARC and facilitate the identification of novel biomarkers and therapeutic targets.

In conclusion, ARC should be recognized as a multifactorial, system-level and drug-specific phenomenon that bridges physiology, pharmacokinetics and critical care medicine. Future progress will depend on consistent renal-function terminology, practical strategies for measuring renal clearance, integration of bedside hemodynamic information, broader implementation of TDM and translational studies that connect experimental models with multi-omics mechanisms.

## Data Availability

Data sharing is not applicable to this article as no new data were created or analyzed in this study.
